# 

**Published:** 1887-11

**Authors:** 


### THE FIRST DISTRICT DENTAL SOCIETY OF THE STATE OF NEW YORK.

Early in the coming January the above Society proposes to hold its nineteenth anniversary.

To those who have attended previous meetings, under the auspices of the First District it is hardly necessary to say that it will, in all probability, be a profitable and pleasant gathering. Every opportunity will be afforded those who attend to see and hear dentistry from a scientific stand-point.

We are creditably informed that the officers are now endeavoring to eclipse their former efforts.

For further information, see Journals for November and December.

### *To Readers and Correspondents!*

Communications intended for publication, and exchange journals and papers should be addressed to the EDITOR OF THE AMERICAN JOURNAL OF DENTAL SCIENCE, 845 N. Eutaw St., Baltimore, Md. All letters relating to the business of the JOURNAL, or containing cash remittances, to be directed to Messrs. SNOWDEN & COWMAN. Articles for publication should be received by the editor at least three weeks previous to the first month on which the number in which it is designed for them to appear, is issued.

☞ The AMERICAN JOURNAL OF DENTAL SCIENCE will contain fifty pages of reading matter in each number, making a volume of about *Six Hundred* pages,

EXCLUSIVE OF ADVERTISEMENTS.

